# Beyond vision: The emotional toll of advanced diabetic retinopathy response to the letter

**DOI:** 10.1111/dme.70296

**Published:** 2026-03-20

**Authors:** Hellena Hailu Habte‐Asres, Clara Nartey, Sarah Afuwape

**Affiliations:** ^1^ Florence Nightingale Faculty of Nursing, Midwifery and Palliative Care King's College London London UK; ^2^ Royal Free London NHS Foundation Trust London UK; ^3^ Moorfields Eye Hospital, NHS Foundation Trust London UK; ^4^ Department of Renal Medicine UCL London UK

Dear Editor,

We thank Gui et al. for their thoughtful correspondence regarding our recent paper, ‘Beyond vision: The emotional toll of advanced diabetic retinopathy’.^1^ We appreciate their engagement with our work and their emphasis on the importance of inclusive psychosocial research among people living with advanced diabetic retinopathy (DR).

We agree that diabetes‐related cognitive impairment is common among individuals with long‐standing diabetes and that emotional distress may persist even in the presence of cognitive decline. Their commentary highlights an important and under‐recognised methodological challenge in psychosocial research involving clinically complex populations.

We were mindful that people attending specialist diabetic retinopathy clinics often experience heightened anxiety related to diagnosis, disease progression and treatment decisions. In this context, we sought to minimise participant burden and avoid introducing additional distress by administering more complex or cognitively demanding assessments during routine clinical encounters.

Notwithstanding this, we wish to clarify that although our study protocol specified exclusion criteria relating to cognitive impairment that could potentially preclude engagement with study procedures, no individuals were excluded from the study on this basis. All individuals who attended the specialist DR clinics during the recruitment period and who consented to participate completed the DDS‐17 questionnaire and were included in the analysis. Consequently, it is unlikely that this approach introduced selection bias related to cognitive status.

The use of validated self‐report instruments, including the DDS‐17, was guided by their established validation parameters, which require a degree of comprehension and self‐reflection to ensure meaningful and interpretable responses. This approach is consistent with the existing literature and reflects common practice in psychosocial research.

We welcome the authors' proposal of a stratified, multimodal assessment framework incorporating cognitive screening, adapted tools and proxy or clinician‐rated measures. Such an approach is methodologically robust and ethically sound and represents an important direction for future research.

Future studies would benefit from prospectively incorporating these inclusive strategies, alongside clearly defined analytical plans to account for data derived from multiple sources. This would strengthen the evidence base and support the development of psychosocial interventions that are responsive to the needs of cognitively vulnerable individuals with advanced DR.
